# The role of DNA hydroxymethylation and TET enzymes in placental development and pregnancy outcome

**DOI:** 10.1186/s13148-023-01483-z

**Published:** 2023-04-25

**Authors:** Sara Vasconcelos, Carla Caniçais, Susana M. Chuva de Sousa Lopes, C. Joana Marques, Sofia Dória

**Affiliations:** 1 Genetics Unit, Department of Pathology, Faculty of Medicine, University of Porto (FMUP), Porto, Portugal; 2 i3S - Instituto de Investigação e Inovação em Saúde, Porto, Portugal; 3 ICBAS-School of Medicine and Biomedical Sciences, University of Porto, Porto, Portugal; 4 Department of Anatomy and Embryology, Leiden University Medical Center, Leiden, The Netherlands

**Keywords:** Epigenetics, Placenta, TET enzymes, 5-hydroxymethylcytosine, Intrauterine growth restriction, Preeclampsia, Pregnancy Loss

## Abstract

The placenta is a temporary organ that is essential for supporting mammalian embryo and fetal development. Understanding the molecular mechanisms underlying trophoblast differentiation and placental function may contribute to improving the diagnosis and treatment of obstetric complications. Epigenetics plays a significant role in the regulation of gene expression, particularly at imprinted genes, which are fundamental in the control of placental development. The Ten-Eleven-Translocation enzymes are part of the epigenetic machinery, converting 5-methylcytosine (5mC) into 5-hydroxymethylcytosine (5hmC). DNA hydroxymethylation is thought to act as an intermediate in the DNA demethylation mechanism and potentially be a stable and functionally relevant epigenetic mark on its own. The role of DNA hydroxymethylation during differentiation and development of the placenta is not fully understood but increasing knowledge in this field will help to evaluate its potential role in pregnancy complications. This review focuses on DNA hydroxymethylation and its epigenetic regulators in human and mouse placental development and function. Additionally, we address 5hmC in the context of genomic imprinting mechanism and in pregnancy complications, such as intrauterine growth restriction, preeclampsia and pregnancy loss. The cumulative findings show that DNA hydroxymethylation might be important for the control of gene expression in the placenta and suggest a dynamic role in the differentiation of trophoblast cell types during gestation.

## Introduction

In mammals, the placenta is a temporary organ that supports fetal development during gestation, mediating the exchange of nutrients, oxygen and waste products between the fetus and the mother, as well as protecting against infections and maternal diseases [1, 2]. These essential functions are only possible due to the complex and chimeric nature of the placenta, which contains cells of both maternal and fetal origin [1, 3].

Up to the blastocyst stage, mouse and human embryos are morphologically similar [4]. In both species, blastocyst implantation is an invasive process, whereupon the trophoblast layer contacts with the uterine cell lining, penetrating deeply into the uterine stroma and ultimately establishing direct contact between the trophoblast-derived cells and the maternal blood. Indeed, this process is a major characteristic that mouse and human share during placental development, both having a hemochorial placenta. However, mouse and human placental development also have striking differences, at the structural and molecular levels [4–6], as described below.

**Fig. 1:**
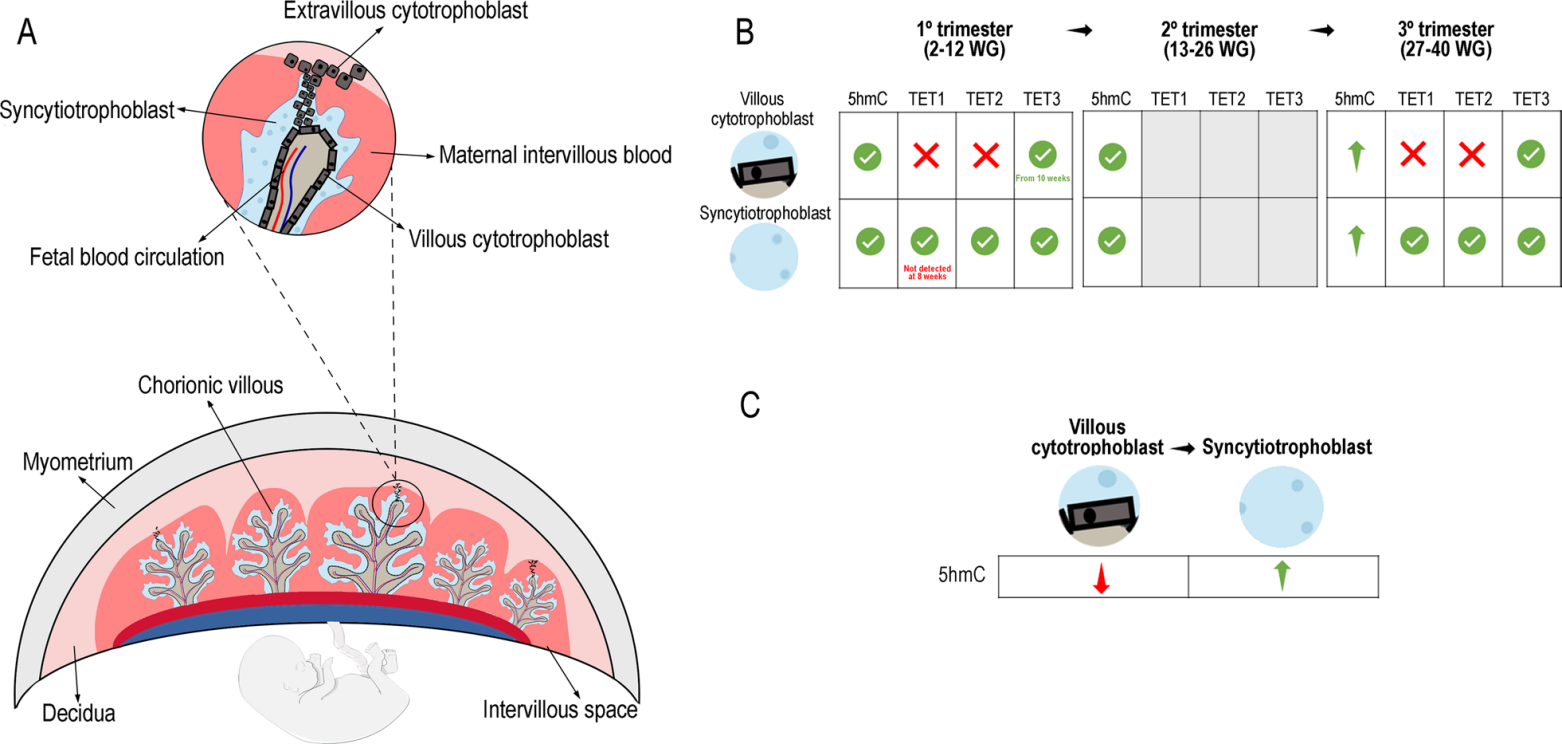
**A** Structure of human placenta (note: drawings are not up to scale). The different types of cells are shown in detail. **B** Comparison of 5hmC and TETs levels between the three trimesters of gestation. **C** Comparison of 5hmC levels between cytotrophoblast and syncytiotrophoblast cells. Green check mark: presence of 5hmC or epigenetic regulator expressed; Red X mark: 5hmC or expression of epigenetic regulator not present; Green arrow: 5hmC or epigenetic regulator levels increased; Red arrow: 5hmC or epigenetic regulator levels decrease; Grey square: not studied

The placenta is a highly organized organ composed of different cell types with specialized functions. During human placental development, the migration of mononuclear cytotrophoblast (CTBs) cells into the decidual space demarcates the beginning of the formation of the primary placental villi. These CTBs differentiate into extravillous CTBs, which have invasive properties and are involved in the implantation process. In addition, these CTBs also differentiate into villous CTBs that fuse and subsequently form the syncytiotrophoblast (STBs) cells, which form a continuous syncytial layer on the surface of the villous tree and contacts directly with the maternal blood flowing through the intervillous space allowing the maternal–fetal exchanges [4, 7]. Throughout pregnancy, the STBs cells are maintained by continuous fusion of the underlying CTBs cells, and therefore, a range of nuclear morphologies with different degrees of chromatin condensation is observed within the STBs cells. Although most nuclei are dispersed within the syncytioplasm, others are aggregates at the surface of terminal villi, referred to as syncytial knots [8]. Therefore, villous structure is formed by a mesenchymal core that contains fetal blood vessels, a layer of CTBs and an outer layer of STBs (Fig. 1A) [4].

**Fig. 2:**
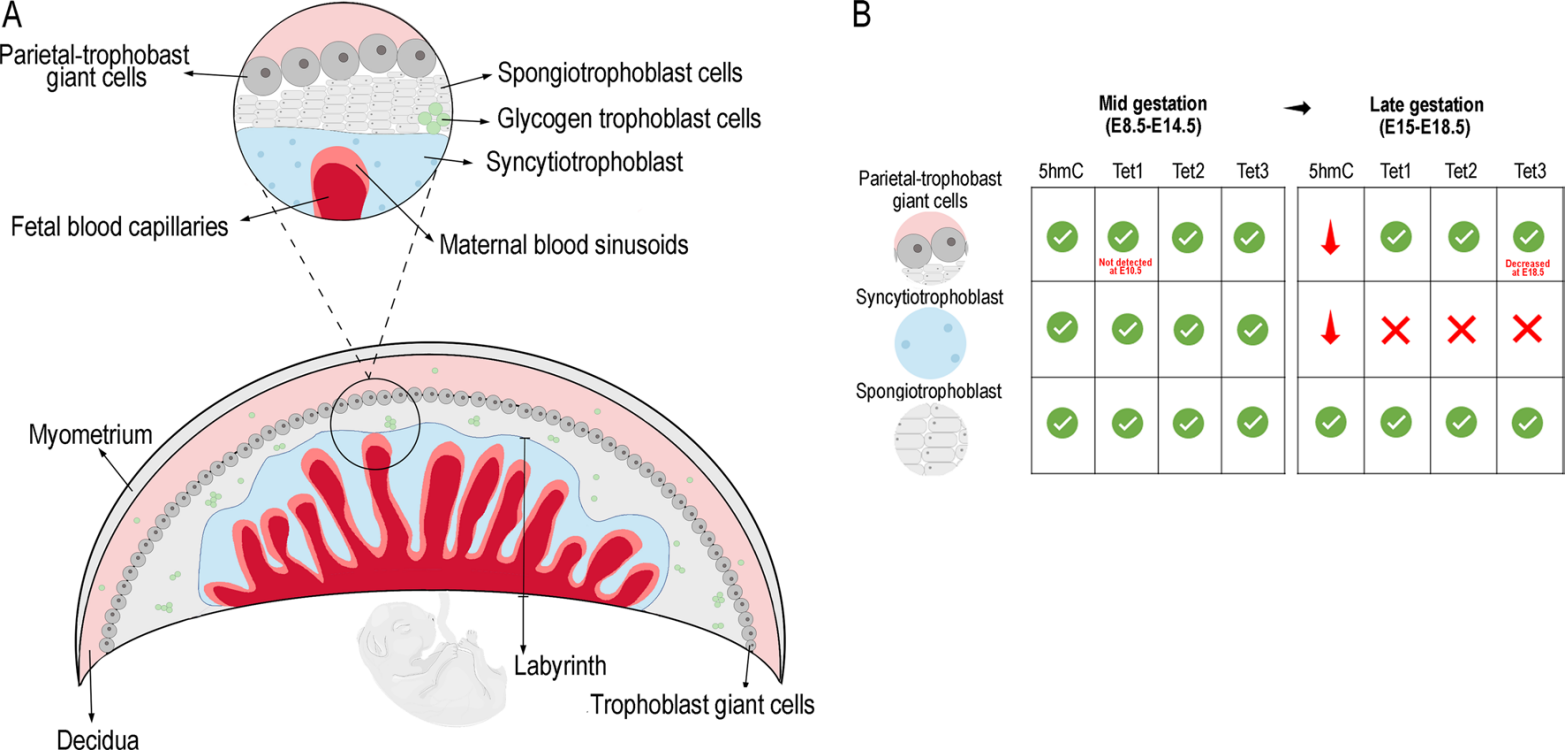
**A** Structure of mouse placenta (note: drawings are not up to scale). The different types of cells are shown in detail. **B** Comparison of 5hmC and TETs levels between mid and late gestation. Green check mark: presence of 5hmC or epigenetic regulator expressed; Red X mark: 5hmC or expression of epigenetic regulator not present; Green arrow: 5hmC or epigenetic regulator levels increased; Red arrow: 5hmC or epigenetic regulator levels decreased

The equivalent in the mouse to the human villous placenta, where the fetal-maternal exchange takes place, is known as the labyrinth, which has a complex arrangement of maternal and fetal vascular channels [9]. Above the labyrinth, there is a junctional zone that consists of trophoblast giant cells (TGCs), spongiotrophoblasts (SpT), and glycogen trophoblast cells (GlyT) that have endocrine functions **(**Fig. 2A**)** [4, 10].

Defective placentation, during trophoblast differentiation and/or invasion, can affect embryonic and fetal development, resulting in pregnancy complications, such as preeclampsia (PE) and intrauterine growth restriction (IUGR), which can ultimately result in spontaneous pregnancy loss [11–13]. Hence, investigating the molecular mechanisms governing placental development throughout gestation is essential to predict pregnancy outcomes.

There is a growing interest in understanding the role of epigenetics in the regulation of placental development. DNA methylation is the best well-studied epigenetic mark and consists on the transfer of a methyl group (-CH_3_), from S-adenosyl methionine (SAM) donor, onto the fifth carbon of a cytosine to form a 5-methylcytosine (5mC) [14]. This reaction is catalyzed by the DNA methyltransferases (DNMTs), which can be divided into de novo methyltransferases (DNMT3A, DNMT3B and DNMT3L, a co-factor for DNMT3A and DNMT3B) and maintenance methyltransferase (DNMT1). The DNMT3A and DNMT3B are capable of methylating ‘naked’ DNA, whereas DNMT1 maintains the DNA methylation pattern during DNA replication in mitotic cell divisions by copying to the newly synthesized DNA strand [15–17]. A high density of methylated CpGs at gene promoter regions usually results in gene silencing due to three-dimensional changes in chromatin conformation, that limit the accessibility of the transcriptional start site (TSS) to the transcriptional machinery and relevant transcription factors [14]. On the other hand, Ten-Eleven-Translocation enzymes (TETs) catalyze the oxidation of a pre-existing 5mC into 5-hydroxymethylcytosine (5hmC), resulting in a less explored epigenetic modification, DNA hydroxymethylation [18, 19]. Mammalian TET family members (TET1, TET2 and TET3) mediate the oxidation reactions, requiring Fe(II) as a cofactor, as well as oxygen and α-ketoglutarate as substrates [20]. The TETs also convert 5hmC into 5-formylcytosine (5fC) and 5-carboxylcytosine (5caC), which act as intermediates in the active DNA demethylation process [21]. Indeed, 5hmC might be more than an intermediate in the active DNA demethylation pathway, playing biological functions and being a relatively stable epigenetic mark, particularly in the nervous system where it is most abundant [22, 23]. Additionally, it has been reported that 5hmC is enriched at genomic imprinted regions in the placenta [24] and imprinted genes are known to play crucial roles in placentation and fetal growth [25].

## TET enzymes and DNA hydroxymethylation in placental development

TET enzymes are involved in the differentiation and regulation of placental trophoblasts [26] and a dysregulation of 5hmC levels can explain an abnormal development of placenta. There are a limited number of studies addressing the role of TET enzymes and DNA hydroxymethylation in placental development. It has been described that 5hmC levels across the placental genome are notably lower than 5mC levels, as expected and observed in other tissues; nevertheless, the existence of CpG sites with consistently elevated levels of 5hmC, above a specific threshold and reproducible between the samples, have been described in the following two studies. Green and colleagues observed ~ 21,000 sites showing a median 5hmC value of 13.73% whereas Mora and colleagues detected around 17,000 CpG sites with 5hmC enrichment [24, 27]. Both studies observed that 5hmC is enriched in open sea (isolated CpGs in the genome) and shelf (2–4 kb distant from CpG islands) regions and depleted in CpG islands. Additionally, they observed enrichment in gene bodies and in 5’ and 3’-UTRs, and depletion in proximal promoters (~ 200–1500 bp from TSS) and at the TSS. Moreover, Green and colleagues reported enrichment within poised enhancers and depletion within active enhancers whereas Mora and colleagues reported 5hmC in imprinted genes such as *GNAS* and *H19* and in placenta-specific DMRs, often with overlapping in regions associated with parent of origin allelic 5mC. Strikingly, Green and colleagues reported an elevation of both 5mC and 5hmC in transcriptionally inactive genes, when compared with actively transcribed genes, wherein 5hmC was positively associated with transcription. These apparently contradictory observations might be the result of an increased turnover rate that is related to the elevated methylation levels observed in transcriptionally inactive genes that translate into increased hydroxymethylation. 5hmC was also more likely to occur in transitional genes, i.e., genes that were differentially expressed between the second trimester and term placentas [27].

In the human placenta, Fogarty and colleagues reported that 5hmC was more abundant in STBs compared to CTBs cells, with 20–50% of STB nuclei in first and second trimesters and 50% in the third trimester staining positively for 5hmC, particularly in syncytial knots consisting entirely of 5hmC positive nuclei; in contrast, the majority of STB nuclei do not display appreciable levels of 5mC staining across gestation while over 50% of CTBs nuclei are 5mC positive [28]. Using immunofluorescence, Wilson and colleagues showed an increase of 5hmC (and 5mC) in both CTBs and STBs cells from term placentas when compared to first and second trimester placentas (Fig. 1B). Additionally, the authors also observed that in the first and second trimesters, 5hmC staining was greater in STBs compared to CTBs (Fig. 1C) [29]. Throughout gestation, with the fusion of CTBs giving rise to STBs, there seems to be an accumulation of 5hmC in the STBs, particularly in the syncytial knots, which might reflect oxidative damage that occurs in the knot formation [28]. These results suggested differences between the two cell types that might reflect their different functions; for example, villous trophoblast regulates gas and nutrient exchange whereas the STBs produces and secretes very large quantities of hormones throughout pregnancy, revealing the high transcriptional and translational capacities of this tissue. Additionally, CTBs and STBs have their unique transcriptome profile, as reported by Rouault et al. [30].

In the mouse placenta, the ectoplacental cone cells (EPCs) and parietal-TGCs exhibited strong 5hmC and lower 5mC levels, at embryonic day (E)8.5. Nuclear 5mC levels were maintained in all labyrinth cells from E10.5-E18.5, with the STBs nuclei showing high 5mC levels throughout gestation. At the same time, high 5hmC levels were present in all labyrinth trophoblast cells at E10.5 but levels gradually decreased by E18.5. In addition, parietal-TGCs showed gradual decrease in 5hmC, while 5mC levels steadily increased at E18.5 (Fig. 2B) [31]. Altogether, these studies highlighted that the epigenome of the different trophoblast-derived cell types is dynamic and subject to changes during gestation.

On the other hand, information about the function of TET enzymes during trophoblast differentiation is scarce. In fact, knockdown models of these enzymes have shown that 5hmC is essential for maintaining development and early embryogenesis [32]. Yamaguchi and colleagues showed that paternal Tet1 KO mice resulted in several phenotypes, including placental defects. Indeed, the authors observed that placental sizes of paternal Tet1 KO mice were significantly smaller compared with controls [33]. Rakoczy and colleagues studied the expression of TET enzymes in mouse and human placentas. In mouse, they observed that *Tet1, Tet2 and Tet3* mRNAs were expressed in chorion, EPCs and yolk sac at E8.5, and in the labyrinth (include sinusoidal-TGCs and STBs cells) at E10.5 to E12.5. However, *Tet1-3* expression became restricted to sinusoidal-TGCs at E14.5 to E18.5 and *Tets* expression was not detected in the STBs, which is in agreement with the levels of 5hmC observed in the STBs at late gestation. Regarding to SpT cells, *Tet1-3* were constantly expressed during all gestation, while in GlyT cells were only apparent between E14.5-E16.5 and were strongly expressed. Additionally, parietal-TGCs expressed all *Tets* during all stages of gestation, except at E10.5 and at E18.5 that occurred an absence of *Tet1* expression and a decrease of *Tet3* expression, respectively (Fig. 2B) [31]. Thus, the decreased levels of 5hmC in parietal-TGCs were concordant with the decrease of *Tet3* expression at E18.5. In the human placenta, TET1-3 proteins were detected in STBs cells at first and third trimesters, with the exception at 8 weeks of gestation (WG), where TET1 was undetectable. Villous CTBs cells do not expressed TET1 and TET2 in the first and third trimesters; however, TET3 was expressed from 10WG until term (Fig. 1B) [31]. Therefore, these results suggested that TET1 and TET2 have a critical role in STBs differentiation. Additionally, the higher levels of 5hmC in STBs compared to CTBs may be result of the expression of all three TETs in STBs, whereas only TET3 is present in CTBs. Since placental development is a highly dynamic process, it would also be important to study how these enzymes are expressed in placentas from second trimester.

Concerning non-catalytic roles of TET enzymes, it has been shown that they interact with some epigenetic modifiers such as histone-modifying enzymes, leading to repression of TET-targeted genes [34]. One example is the interaction observed between TET1 and EZH2 [35], which is responsible for establishment of H3K27me3, a histone mark that is highly prevalent in CTB nuclei, along with other histones modifications [28].

Although there are differences in placental structure regarding types of cells, both 5mC and 5hmC, as well as TETs expression, were observed in mouse and human trophoblast cells during gestation. However, an exception may be observed at the end of gestation regarding STBs, since expression of TETs was not observed in mouse but were observed in humans. Besides that, in human, an increase of 5hmC levels was observed along the gestation, while in mouse it was observed a decrease. Nevertheless, more studies are needed to confirm the differences observed between mouse and human, as well as to clarify its role in cell regulation and differentiation during placental development.

## DNA hydroxymethylation in placental imprinted genes

Genomic imprinting was first described in 1984 after nuclear transplantation experiments showing that mouse embryos with two sets of maternal chromosomes (gynogenotes) or two sets of paternal chromosomes (androgenotes) failed to develop normally, with the first showing growth retardation and abnormal development of the extra-embryonic tissues (placenta and yolk sac) and the latter showing overgrowth of extra-embryonic tissues and poor-development of the embryo proper, indicating that, despite having similar genetic information, the inherited maternal and paternal chromosomes were not functionally equivalent [36, 37]. Genomic imprinting is an epigenetic mechanism whereby imprinted genes show a monoallelic and parent-of-origin-dependent expression, regulated by differentially methylated regions (DMRs) in the DNA. Some of these DMRs act as imprinting control regions (ICR) and control the expression of imprinted genes clusters, that can harbour several adjacent maternally-imprinted and paternally-imprinted genes (as reviewed in [38]). Therefore, the deregulation of imprinted genes expression may affect the normal development of the fetus and result in human diseases, such as imprinting syndromes [39].

Although DNA methylation has been extensively studied in the context of genomic imprinting, the role of DNA hydroxymethylation on the regulation of ICRs and, consequently, in the expression of imprinted genes, has been less studied. Indeed, studies showed that double *Tet1*/*Tet2* knockout mice had abnormal DNA methylation at various imprinted loci [40]. Additionally, the involvement of *Tet1* and *Tet2* in the efficient erasure of genomic imprints in a cell fusion model [41] and the involvement of TET1 and TET2 in imprint erasure in mouse primordial germ cells via conversion of 5mC into 5hmC [42] pointed to a crucial role for 5hmC in erasure, establishment and maintenance of genomic imprinting. However, the knowledge about the effects of TETs deficient in the placenta-specific imprinted genes is still scarce. TET1 has a critical role in the erasure of paternal imprints, since hypermethylation patterns have been observed in several DMRs of paternally expressed genes, such as *Peg10* and *Peg3*, in sperm from *Tet1* deficient mice [33]. Additionally, *Peg3* DMR hypermethylation was observed in *Tet1* knockout placentas. This led to a number of variable phenotypes such as placental, fetal and postnatal growth defects and early embryonic lethality.

In human placentas, an association between the expression of the imprinted gene *CDKN1C* and birth weight and 5hmC enrichment at the ICR controlling the expression of *CDKN1C* has been shown [43]. Hernandez-Mora and colleagues also detected monoallelic 5hmC enrichment at the *GNAS* A/B DMR, *H19* gene body and several placenta-specific DMRs, such as *MCCC1*, *RHOBTB3*, *SCIN*, *DNMT1*, and *ACTL10*. Some of the genes that show 5hmC enrichment at the DMR have been associated with imprinting diseases [44], hence the study of 5hmC levels at these DMRs is of particular relevance. Additionally, 5hmC was found solely on the methylated allele of imprinted DMRs [24], as would be expected considering that 5hmC results from the oxidation of previously methylated DNA.

## DNA hydroxymethylation in intrauterine growth restriction (IUGR)

IUGR is a complex and common obstetric complication in which the fetus fails to achieve its growth potential and is associated with significant perinatal morbidity and mortality. The classification of IUGR is based on estimated fetal length by ultrasound measurements, when below 10th percentile for gestational age [45]. The causes of IUGR might be of maternal, placental or fetal origin, with placental insufficiency being one of the main causes due to deficiencies in nutrient and oxygen transportation across the placenta [46].

In addition to the extensive study of DNA methylation in IUGR placentas [47], DNA hydroxymethylation has also been analyzed in IUGR placentas (summarized in Table 1). The study of monozygotic (MZ) twins is valuable in understanding the epigenetic basis of pregnancy complications and human diseases. The decrease in global DNA hydroxymethylation in selective intrauterine growth restriction (sIUGR) placentas was described in a study of MZ twins [48]. Similarly, Zhang and colleagues also observed a decrease in global 5hmC levels in the smaller placental share of sIUGR pregnancies [49]. Additionally, the authors observed lower levels of 5hmC in the promoters of *ANGPTL4* and *HIF1A* genes, which are hypoxia-responsive genes that were downregulated in the smaller placental share of sIUGR [49]. This may occur because TET enzymes require oxygen to oxidize 5mC into 5hmC [50]. Although the authors did not directly measure hypoxia state and TET activity at the placental share corresponding to the fetus with restricted growth, experimental evidence in the animal model has shown that in utero hypoxia resulted in fetal growth restriction [51]. Additionally, studies in cancer have shown that hypoxia influence the activity of TET enzymes [52]. Our group also studied the expression of epigenetic regulators in IUGR placentas and observed an increase in *TET3* expression, as well as all three *DNMT*s (*DNMT1*, *3A* and *3B*); however, no changes in global levels of DNA methylation or hydroxymethylation were observed [53]. These findings highlight a potential contribution of DNA hydroxymethylation in discordant fetal growth or even placenta-associated disorders.

**Table 1:** DNA hydroxymethylation studies in placentas associated with pregnancy complications, such as intrauterine growth restriction, preeclampsia, and pregnancy loss.

Pathology	Species	Gestational age	Sampling location	Number of samples	Methodology	Findings	Ref
IUGR	Rat	–	–	IUGR: *n* = 12 non-IUGR: *n* = 12	Gluc-MS-qPCR; RT-qPCR	In IUGR rat, induced by gestational protein deficiency, the levels of 5hmC in the *Wnt2* promoter and the *Wnt2* expression were decreased	54
	Human	sIUGR twins: 31–35 weeks	Placental villous tissues around umbilical cord insertion region	19 pairs of sIUGR twins	UPLC-MS/MS	Decrease in global DNA hydroxymethylation in sIUGR placentas	48
	Rat	16 days	–	Rats exposed to TCE: *n* = 8 Controls: *n* = 7	5-hmC DNA ELISA; RT-qPCR	Fetal weight is negatively influenced by exposure to maternal TCE. Placentas of rats exposed to TCE have increased 5hmC global levels and increased expression of *Tet3*	55
	Human	sIUGR twins: 32.58 ± 2.19 weeks Normal MC twins: 36.02 ± 1.17 weeks	Placental villous tissues around umbilical cord insertion region	13 pairs of sIUGR twins 18 pairs of normal MC twins	hMeDIP-qPCR; Dot blot; IHC	Decreased global levels of 5hmC in sIUGR placentas Decreased *ANGPTL4* expression and *ANGPTL4* promoter with aberrant levels of 5hmC under hypoxic conditions	49
	Human	IUGR: 37.12 ± 1.10 weeks non-IUGR: 38.54 ± 1.26 weeks	Placental villous tissues around umbilical cord insertion region	IUGR: *n* = 21 non-IUGR: *n* = 9	5-hmC DNA ELISA; RT-qPCR	No differences in global DNA hydromethylation were detected between IUGR and non-IUGR samples IUGR placentas showed increased *TET3* expression	53
Preeclampsia	Human	PE: 36 ± 0,5 weeks non-PE: 37.3 ± 0.4 weeks	Placental tissue from the center of the maternal side	PE: n = 21 non-PE: *n* = 16	Western blot; Dot blot; IHC	PE placentas showed that 5hmC global levels and TETs expression were decreased	60
	Human	PE: 38.25 ± 0.85 weeks non-PE: 38.62 ± 0.63 weeks	–	PE: *n* = 20 non-PE: *n* = 20	hMeDIP-seq; (h)MeDIP-qPCR	Severe PE and non-PE samples showed genome-wide mapping of 5hmC differences however, no differences in global DNA hydroxymethylation were observed	63
	Human	PE: 35.16(30.29–39) weeks non-PE: 39.49(38.86–40.86) weeks	Placental tissue from the maternal side, around the central villus area	PE: *n* = 13 non-PE: *n* = 11	RT-qPCR; Western blot; Dot blot; IHC	5hmC levels and TET2 mRNA and protein expression are decreased in PE placentas	61
	Human	PE: 37.27 ± 0.36 non-PE: 38.89 ± 0.13 weeks	Placental tissue from the center of the maternal side	PE: *n* = 10 non-PE: *n* = 10	RT-qPCR; IHC; Western blot.; IHC; ELISA	Global 5hmC and TET expression are decreased in PE placentas and hypoxic trophoblast	62
	Human	PE: 39.33 ± 1.37 non-PE: 40.65 ± 0.45 weeks	–	PE: *n* = 10 non-PE: *n* = 9	IHC	No differences in 5hmC levels between PE placentas and non-PE. However, the levels of 5hmC are different across gestation and between different placental cell types	29
Pregnancy loss	Human	6–8 weeks	Chorionic villi	Early PL: *n* = 100 (6 weeks, *n* = 27; 7 weeks, *n* = 35; 8 weeks, *n* = 38) and normal pregnancy: *n* = 126 (6 weeks, *n* = 36; 7 weeks, *n* = 40; 8 weeks, *n* = 50)	RT-qPCR; Dot-blot; Western Blot; IHC	Expression of TETs and 5hmC in normal villus decreased with increasing gestational age. TET1-3 expression was lower in the early PL group than normal pregnancy group	71
	Human	12–24 weeks	Chorionic villi	Placenta controls: *n* = 16 Idiopathic PL: *n* = 19	RT-qPCR; ELISA assay	Upregulation of *TET2* and *TET3* in placental samples from idiopathic PL; No significant difference in global 5hmC levels was observed	72

*ANGPTL4* Angiopoietin Like 4; *hMeDIP-seq* Hydroxymethylated DNA immunoprecipitation sequencing; *(h)MeDIP-qPCR* Hydroxymethylated DNA Immunoprecipitation quantitative PCR; *IUGR* Intrauterine growth restriction; *IHC* Immunohistochemistry; *MC* monochorionic; *PE* Preeclampsia; *PL* Pregnancy loss; *RT-qPCR* quantitative real-time PCR; *sIUGR* selective intrauterine growth restriction; *TCE* trichloroethylene; *TET* Ten-eleven translocation; *UPLC-MS/MS* ultraperformance liquid chromatography-tandem mass spectrometry; *Wnt2* Wnt Family Member 2; *5hmC* 5-hydroxymethylcytosine

It is also important to have in consideration that environmental conditions may influence the placental epigenome and may affect fetal growth. Experiments in rats showed that administration of a low protein diet or maternal exposure to trichloroethylene, a widespread environmental pollutant, during gestation led to the deregulation of 5hmC levels and was associated with fetal growth restriction [54, 55]. In this IUGR rat model, 5hmC levels in the *Wnt2* promoter and *Wnt2* gene expression were reduced, suggesting that nutrient deficiency, that culminates in IUGR, might change the epigenetic state of the placenta [54]. Furthermore, it was observed that maternal exposure to trichloroethylene negatively influenced rat fetal weight during mid-pregnancy and increased the 5hmC levels and *Tet3* expression [55].

## DNA hydroxymethylation in preeclampsia

Preeclampsia (PE) is a pregnancy-associated disorder that affects about 3–5% of pregnant women and is characterized by the onset of hypertension and proteinuria after 20WG in a previously normotensive woman [56]. This pathology may lead to maternal, perinatal and fetal mortality and morbidity and is linked with IUGR, placental abruption, preterm birth, and long-term complications such as cardiovascular diseases [57].

Several epigenetic changes have been associated with PE, such as DNA methylation and hydroxymethylation modifications [58]. One study showed hypermethylation at the promoter region of *H19,* with consequently decreased mRNA expression in early-onset preeclamptic placentas [59]. In this study, the authors also showed an increase in global DNA methylation levels and in *DNMT1* methyltransferase mRNA. More recently, several studies also addressed the levels of DNA hydroxymethylation in PE placentas (Table 1). Liu and colleagues observed a decrease in global 5hmC levels, concordant with a decreased expression of *TET1-3* in PE placentas [60]. Other studies also reported lower levels of global 5hmC and decreased TET levels in PE placentas, when compared with control placentas [61, 62]. Li and colleagues observed a decrease of the 5hmC levels and *TET2* transcripts in the PE placentas. This was concordant with the results obtained when employed a TET2 shRNA knockdown cellular model, where observed that TET2 downregulation led to a decrease in 5hmC levels and a reduction in trophoblast migration and invasion [61]. Additionally, the authors observed a downregulation of *TET2* that inhibited *MMP9* expression, an angiogenesis-related protein, which could be rescued by vitamin C, an activator of TET enzyme activity. Ma and colleagues showed that *IGF1* mRNA and protein expression were decreased in PE placentas, as well as in hypoxic-cultured cells [62]. Furthermore, PE placentas and trophoblast cultured under hypoxic conditions showed decreased levels of global 5hmC and increased levels of global 5mC. This decrease of 5hmC was consistent with the decrease of TETs expression observed in PE placentas. On the other hand, other study did not observe differences in 5hmC global levels between PE placentas and placentas from uncomplicated pregnancies, although levels of 5mC were higher in PE placentas [29]. Zhu and colleagues also did not observe differences in global levels of DNA hydroxymethylation, as well as in global levels of DNA methylation, between PE and non-PE placentas. However, locus-specific modifications were detected, including 714 DMRs, that were associated with 403 genes, and 119 differentially hydroxymethylated regions (DhMRs), overlapping 61 genes. Furthermore, the regions studied did not have simultaneous changes in 5mC and 5hmC, highlighting that both epigenetic marks may have an independent role in late-onset severe PE [63].

Most of the studies using Dot blot and IHC showed lower 5hmC global levels in PE samples (Table 1); however, a study using a more informative technique (hMeDIP-Seq) did not detect genome-wide differences albeit describing locus-specific differences. The point of sampling might also interfere with the results although most studies referred to have analyzed placental tissues from the maternal side.

## DNA hydroxymethylation in pregnancy loss

Pregnancy loss is one of the most common obstetric complications that has a strong clinical and social impact. Indeed, studies have demonstrated an association between early pregnancy loss and a higher rate of psychological morbidity, such as depression, anxiety and post-traumatic stress symptoms [64]. About 10–15% of clinically recognized pregnancies terminate in a spontaneous pregnancy loss and this frequency is similar among various human populations [65, 66]. Pregnancy loss can be a sporadic or a recurrent event, and approximately 1–5% of the cases correspond to recurrent pregnancy losses [67]. This clinical complication has a heterogeneous etiology, resulting from several known risk factors, such as uterine anatomic defects, immunological, infectious, environmental, endocrine, thrombophilic, and genetic factors [68, 69]. Despite these different causes, genetic factors are considered the main cause of pregnancy loss [65], with fetal chromosomal abnormalities representing 50–60% of the cases [66]. However, a significant proportion of the recurrent pregnancy losses have a normal karyotype and remain idiopathic.

Since the placenta plays a role in maternal–fetal exchanges and endocrine function [2], perturbations in the development and function of the placenta can have a negative impact on the fetus and culminate in pregnancy loss [70]. As mentioned above, increasing evidence suggests that epigenetic dysfunction may influence the stability of normal pregnancy and lead to complications during pregnancy, such as IUGR and PE; however, it remains elusive whether dysfunctional TET enzymes and abnormal levels of 5hmC are involved in an abnormal placental function that can result in pregnancy loss (Table 1).

Wu and colleagues studied the expression of TETs and 5hmC levels in chorionic villi from spontaneous early pregnancy losses (EPL) and normal pregnancies (undergoing medical termination of pregnancy) [71]. This study showed that the expression of TET1, TET2 and TET3 was lower in the villi of the EPL group than in the normal pregnancy group, from 6 to 8WG, and 5hmC levels were lower in EPL group, but only at 6WG [71]. Additionally, it was observed that expression of TETs and 5hmC in normal villous decreased with increasing gestational age. Our group also studied *TETs* expression and 5hmC levels in human placentas from spontaneous abortions of second trimester gestations and we also observed a deregulation of *TETs* in placental tissues from idiopathic pregnancy losses [72]. However, we observed an overexpression of *TET2* and *TET3* in placental samples from idiopathic cases, at 12-24WG [72]. Despite *TET2* and *TET3* being upregulated in placentas from idiopathic spontaneous abortions, we did not observe statistically significant changes in global levels of DNA hydroxymethylation [72]. In the future, it would be useful to analyse the levels of 5hmC in specific genomic regions of placental DNA from idiopathic pregnancy loss, such as regulatory regions of imprinted genes that are 5hmC enriched.

## Conclusion

The placenta is essential to support fetal development during pregnancy. Although new techniques, such as single cell RNA sequencing, are now emerging and revealing specific and unique features of human trophoblast cells, it remains challenging to study the complexity of human placenta and the dynamics of placental development. Dysfunctional placental development underlies different pregnancy complications, and for that reason, a detailed understanding of the molecular mechanisms that govern trophoblast differentiation may improve the diagnosis and treatment of these disorders. It is an exciting time for placental research with emerging knowledge on the role that epigenetic mechanisms, particularly DNA hydroxymethylation, may have during human placental development in normal and complicated pregnancies. Thus, DNA hydroxymethylation may be an important epigenetic mechanism for the development of a functional placenta.

In conclusion, the studies support a role for TET and 5hmC in placental development; however, functional studies, downregulating TETs specifically in the placenta, are needed, both in vitro and in the animal model, to help clarify the role of these epigenetic modifiers in placental cells differentiation and function. More studies focused on 5hmC and TETs expression on normal and pathologic placentas, specific diseases such as IUGR, PE and pregnancy loss and also in specific genomic regions namely on regulatory gene regions, TSS or CpG islands should be performed. This will allow increasing the knowledge and potentially contribute to prevent or at least to manage better some obstetric complication.

## Data Availability

Not applicable.
